# GTP Cyclohydrolase I and Tyrosine Hydroxylase Gene Mutations in Familial and Sporadic Dopa-Responsive Dystonia Patients

**DOI:** 10.1371/journal.pone.0065215

**Published:** 2013-06-06

**Authors:** Chunyou Cai, Wentao Shi, Zheng Zeng, Meiyun Zhang, Chao Ling, Lei Chen, Chunquan Cai, Benshu Zhang, Wei-Dong Li

**Affiliations:** 1 Research Center of Basic Medical Sciences, Tianjin Medical University, Tianjin, China; 2 Department of Neurosurgery, Tianjin General Hospital, Tianjin Medical University, Tianjin, China; 3 Department of Neurology, Tianjin People’s Hospital, Tianjin, China; 4 Department of Surgery, Tianjin Children’s Hospital, Tianjin, China; 5 Department of Neurology, Tianjin General Hospital, Tianjin Medical University, Tianjin, China; Centre Hospitalier Universitaire Vaudois (CHUV), Switzerland

## Abstract

Dopa-responsive dystonia (DRD) is a rare inherited dystonia that responds very well to levodopa treatment. Genetic mutations of GTP cyclohydrolase I (*GCH1*) or tyrosine hydroxylase (*TH*) are disease-causing mutations in DRD. To evaluate the genotype-phenotype correlations and diagnostic values of *GCH1* and *TH* mutation screening in DRD patients, we carried out a combined study of familial and sporadic cases in Chinese Han subjects. We collected 23 subjects, 8 patients with DRD, 5 unaffected family members, and 10 sporadic cases. We used PCR to sequence all exons and splicing sites of the *GCH1* and *TH* genes. Three novel heterozygous *GCH1* mutations (Tyr75Cys, Ala98Val, and Ile135Thr) were identified in three DRD pedigrees. We failed to identify any *GCH1* or *TH* mutation in two affected sisters. Three symptom-free male *GCH1* mutation carriers were found in two DRD pedigrees. For those DRD siblings that shared the same *GCH1* mutation, symptoms and age of onset varied. In 10 sporadic cases, only two heterozygous *TH* mutations (Ser19Cys and Gly397Arg) were found in two subjects with unknown pathogenicity. No GCH1 and TH mutation was found in 40 unrelated normal Han Chinese controls. *GCH1* mutation is the main etiology of familial DRD. Three novel *GCH1* mutations were identified in this study. Genetic heterogeneity and incomplete penetrance were quite common in DRD patients, especially in sporadic cases. Genetic screening may help establish the diagnosis of DRD; however, a negative *GCH1* and *TH* mutation test would not exclude the diagnosis.

## Introduction

Dopa-responsive dystonia (DRD), also known as Segawa’s syndrome, was first reported in 1976 [1]. The clinical manifestations of DRD include postural or motor disturbances, generalized or focal dystonia, abnormal gait, and sometimes tremor or writing disturbance[2]–[4]. A significant therapeutic response to levodopa is a diagnostic hallmark of DRD. Mutations in the gene encoding GTP cyclohydrolase I (GCH1) are common in the autosomal dominant form of DRD [5], while autosomal recessive forms of DRD can be caused by mutations of the gene encoding tyrosine hydroxylase (TH) [6]. Treatment with levodopa results in significantly clinical improvement in almost all the patients with *GCH1* mutations, however, response of levodopa treatment in some patients with *TH* mutations was limited [7], [8].

Given the rare incidence of DRD (1 in 10^6^), it is difficult to explore genotype-phenotype correlations in patients. Several studies have shown incomplete penetrance and gender differences in DRD [9]. Although the disease is rare, genetic heterogeneity is quite common in DRD, as well as in other dopamine pathway disorders.

During the past 20 years, we have collected 23 subjects (8 patients with DRD, 5 unaffected members, and 10 sporadic cases). For this study, we sequenced all exons and splicing sites of *GCH1* and *TH* for all 23 individuals. Three novel heterozygous *GCH1* mutations were identified in three DRD pedigrees. In sporadic cases, only two heterozygous *TH* mutations were found.

## Patients and Methods

**Figure 1:**
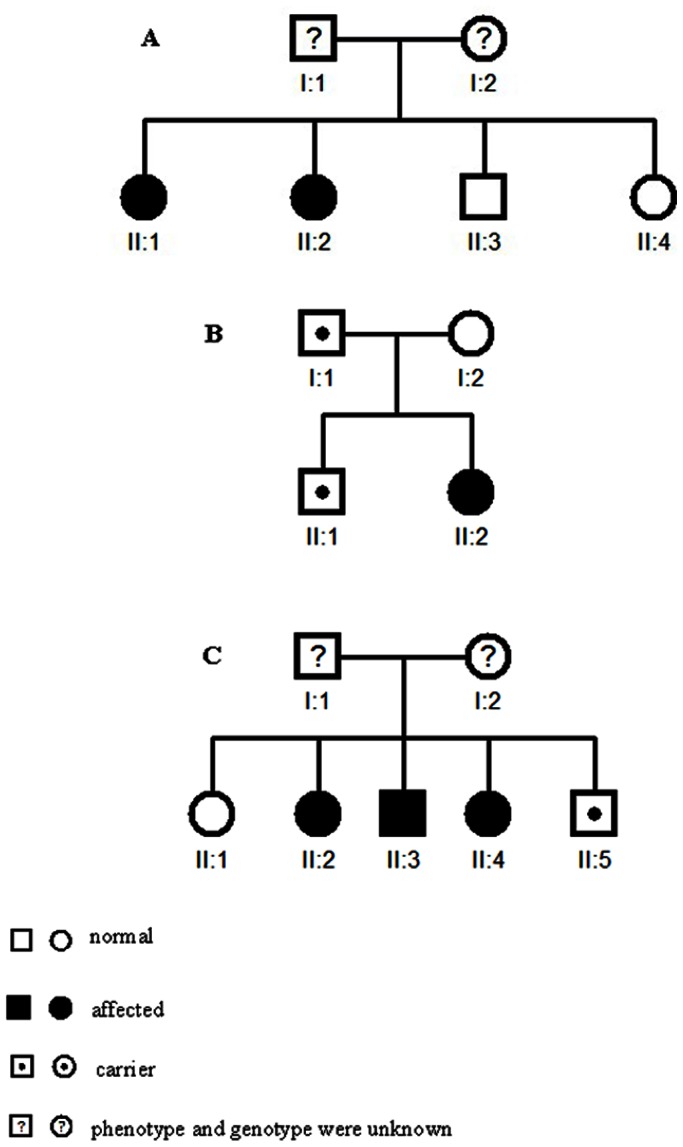
Three DRD pedigrees with *GCH1* mutations: family 12 (A), family 10 (B), and family 9 (C).

DRD patients were diagnosed by criteria suggested by Calne et al. [10] at Tianjin General Hospital. Briefly, all patients had dystonia with marked response to levodopa, most of them with clear patterns of diurnal fluctuation (especially in those with onset before 10 years of age). We collected blood samples from 23 subjects: 8 patients and 5 unaffected family members in three pedigrees (**Figure 1**, **Table 1**), and 10 sporadic patients (**Table 1**). Additionally, 40 unrelated normal Han Chinese controls (20 males and 20 females, age>65 yr) were collected from an ongoing senior citizen cohort study at Tianjin Medical University.

**Table 1:** Clinical characteristics and genetic mutations of GTP cyclohydrolase I (*GCH1*) and/or tyrosine hydroxylase (*TH*) in 23 subjects.

Subject	Family	Sex/age	Age of onset (years)	levodopa dose (mg/day)	Diurnal fluctuation (+/−)	Initial site of dystonia*	Response to levodopa treatment	Neurological signs: Legs affected more than arms (+/−)	GCH1 mutation	THmutation	Other
1	1	F/22	20	375	+	Neck	+	−	−	6127G>A Gly397Arg	Symptoms occur when pregnant
2	2	F/35	10	250	+	Lower extremities	+	+	−	−	Mother experienced mercury poisoning before pregnancy
3	2	F/38	30	375	+	Lower extremities	+	+	−	−	
4	3	F/30	5	375	+	Neck	+	−	−	−	
5	4	M/33	30	375	+	Lower extremities	+	+	−	−	
6	5	F/20	14	−	+	Lower extremities	N/A	+	−	−	Depression No dopa used
7	6	M/22	3	375	+	Right leg	+	+	−	−	
8	7	M/41	13	625	+	Lower extremities	+	+	−	−	
9	8	M/11	10	375	−	Right leg	+	−	−	−	
10	9	F/64	−		−		N/A	N/A	−	−	No symptoms
11	9	M/54	10	125	+	Lower extremities	+	+	454C>T Ala98Val	−	
12	9	F/56	20	125	−	Left leg	+	+	454C>T Ala98Val	−	
13	9	F/50	18	125	−	Right leg	+	+	454C>T Ala98Val	−	
14	9	M/46	−	−	−	−	N/A	N/A	454C>T Ala98Val	−	No symptoms
15	10	F/34	2	187.5	+	Lower extremities	+	+	37449T>C Ile135Thr	−	Grandparents are cousins
16	10	M/36	−	−	−	−	N/A	N/A	37449T>C Ile135Thr	−	No symptoms Brother of patient15 Depression
17	10	M/60	−	−	−	−	N/A	N/A	37449T>C Ile135Thr	−	No symptoms Father of patient15 Depression, alcoholism
18	11	F/48	−	375	−	−	+	−	−	−	
19	12	F/72	60	375	−	−	+	−	385A>G Tyr75Cys	−	
20	12	F/55	15	375	−	−	+	−	385A>G Tyr75Cys	−	
21	12	F/52	−	−	−	−	N/A	N/A	−	−	No symptoms
22	13	F/13	4	375	−	−	+	−	−	75C>G Ser19Cys	
23	14	M/28	22	750	+	Lower extremities	+	+	−	−	Parents are cousins

N/A: not applicable.

All subjects and normal controls gave written informed consent prior to this study, and the protocol was approved by the Committee on Studies Involving Human Beings at Tianjin Medical University.

Clinical characteristics of subjects are given in **Table 1**. The age of onset ranged from 2 to 60 years. We have information of initial sites of DRD onset for 14 patients: 12 were in lower extremities; 2 started in the neck. Almost all patients responded very well to levodopa treatments, although patients with DRD family histories were more sensitive to levodopa.

DNA was extracted from ethylenediaminetetraaceticacid (EDTA)-treated whole blood samples using the standard high-salt method. All exons and splicing sites of *GCH1* and *TH* were amplified by PCR and sequenced by forward and reverse primers. Primers used in PCR and sequencing were designed by Primer3 [11]and are shown in **Table S1**. Sequence alignments were performed by the Mutation Surveyor software (SOFTGENETICS) for mutation detection. Once a mutation was found, 40 normal control subjects were sequenced for that mutation. Three-dimensional structures of the mutant *GCH1* and *TH* proteins were predicted by SWISS-MODEL (http://swissmodel.expasy.org/).

## Results

**Figure 2:**
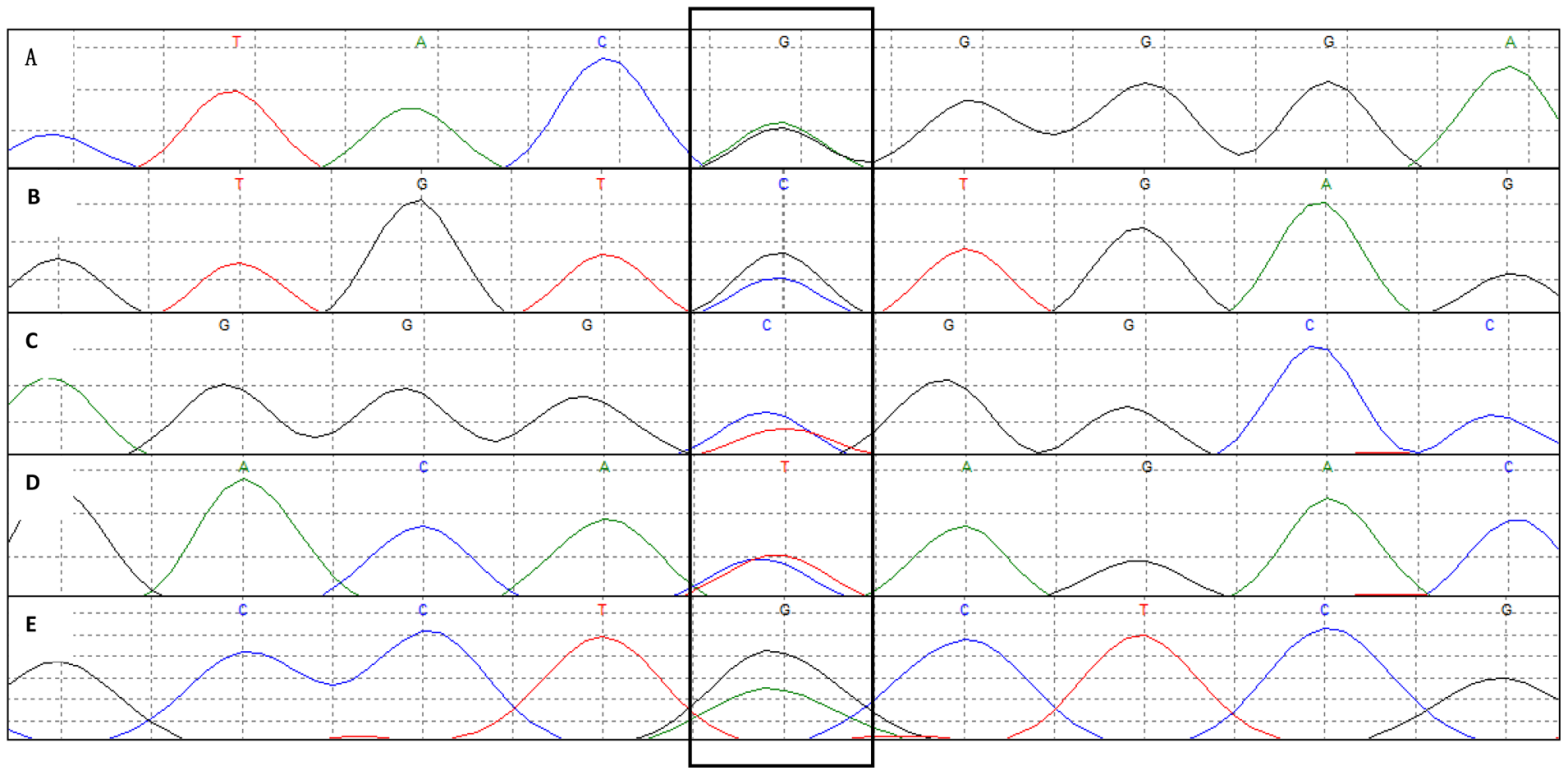
*TH* mutations (A–B) and *GCH1* mutations (C–E). **A** *TH* exon12 mutation of patient 1 (6127 G>A, Gly397Arg). **B**, *TH* exon1 mutation of patient 22 (75 C>G, Ser19Cys). **C**, *GCH1*exon1 mutation of patients 11–14 (454 C>T, Ala98Val). **D**, *GCH1* exon 2 mutation of patient 15 (37449 T>C, Ile135Thr). **E**, *GCH1* Exon1 mutation of patients 19 and 20 (385 A>G, Tyr75Cys). Mutations were shown in the black rectangle.

Three novel heterozygous missense mutations of *GCH1* (Tyr75Cys, Ala98Val, and Ile135Thr ) were found in this study.(**Figure 2**, **Table 1**).

In family 9 (Figure 1C), the 454C>T mutation in exon 1 of *GCH1* changed an alanine to a valine at codon 98. Four of five siblings in this family carry the Ala98Val mutation, but the only male mutation carrier (also the youngest, 46 years of age) had no symptoms of dystonia.

In family 10 (Figure 1B), we found a mutation in exon 2 (37449T>C) that resulted in an isoleucine/threonine transition at codon 135. The proband was a 34-year-old female); her age of onset was 2 years. A mutation at the same position has been found in a French family, but that mutational pattern is T>T/A [12]. The same *GCH1* Ile135Thr mutations were found in the proband’s father and elder brother, although neither was affected.

In family 12 (Figure 1A), we have identified two siblings with heterozygous Tyr75Cys (385A>G) mutations, while the other sibling, who does not carry this mutation, is normal.

Two heterozygous mutations were detected in *TH* exon 12 (Gly397Arg) and exon 1(Ser19Cys) in sporadic DRD cases of our study (**Table 1**, individuals 1 and 22).

As shown in **Table 1**, we found no *GCH1*or *TH* mutations in the other eight sporadic cases. No *GCH1* or *TH* mutation was found in 40 normal controls.

## Discussion

Since *GCH1*mutations were first found in DRD (or Segawa’s syndrome) pedigrees [5], more than 100 *GCH1* mutations have been identified in DRD patients [13]. Many asymptomatic *GCH1* carriers were found, suggesting a moderate penetrance of *GCH1* mutations [9], [14]. In our clinical practice, we have found many “sporadic” DRD cases without any family history. Even among patients with multiple affected siblings, the spectrum of symptoms and age of onset were quite variable. On the other hand, we could not rule out genetic heterogeneity since *TH* mutations might also account for the pathogenesis of this rare disease. To decipher the genotype-phenotype connections among DRD patients, we carried out *GCH1* and *TH* sequencing in 23 subjects, including 13 in DRD pedigrees.

We have identified three novel *GCH1* mutations in DRD families: Tyr75Cys, Ala98Val, and Ile135Thr. The crystal structure of GCH1 suggests that the active site of the enzyme forms a narrow pocket, with the hydrophobic residues 131–139 constituting part of the inner wall of the pocket [15]. The substitution of the hydrophobic residue (isoleucine) by the polar threonine at codon 135 may influence enzyme activity. Two other missense *GCH1* mutations are unlikely to change the protein structure since both the wild-type and mutant alleles code the same category of amino acids. Compared with frame-shift mutations, the three heterozygous mutations that we found in our study are relatively benign: two of these three mutations are unlikely to change protein structure. We cannot rule out the possibility that deletions of the *GCH1* gene, other gene mutations or modifiers might account for DRD in these pedigrees.

Although all three *GCH1* mutations found in this study were mostly co-segregated with the DRD affection status, we needed to find out whether the residue changes were polymorphisms. Orthologies between human, mouse, and rat *GCH1* and *TH* genes were computed by BLASTP (http://blast.ncbi.nlm.nih.gov/). All 5 novel mutations found in this study (Tyr75Cys, Ala98Val, and Ile135Thr in *GCH1*, Ser19Cys and Gly397Arg in *TH*) were conserved in evolution. Moreover, none of these mutations were found in 40 normal individuals. It is highly unlikely that these *GCH1* and *TH* mutations were polymorphisms.

In family 10, we found the *GCH1* Ile135Thr mutation in the unaffected father and brother (**Figure 1B**, **Table 1**, **Figure 2D**), both of whom had depression but not dystonia. An increased frequency of psychiatric dysfunctions, including major depressive, anxiety, and obsessive-compulsive disorders, manifested in a cohort of 18 subjects with *GCH1* deficiency, and reduced levels of 5-hydroxyindolacetic acid and 3-methoxy-4-hydroxyphenylglycol in cerebrospinal fluid have been shown [16], [17]. Four members in another family with a *GCH1* exon2 mutation also had significant psychiatric dysfunction, including depression and anxiety [18].

The ages of onset, symptoms, and reactions to treatment were quite different for individuals that shared the same *GCH1* mutation. Three adult male *GCH1* mutation carriers in two families showed no symptoms of dystonia, although their female siblings/offspring had relatively severe DRD. A study of five DRD families demonstrated significant variations in expressivity, even among affected members of the same pedigree [19]. In our study, we found that there is indeed marked intrafamilial variability in age of onset, including a 45-year gap between two affected siblings in family 12.

Previous research has demonstrated a sex bias in DRD patients, with a female:male ratio of 4.3; the *GCH1* mutation penetrance is 2.3 times higher in females than in males [9]. In the present study, three male *GCH1* mutation carriers had no symptoms of dystonia, although all of them were older than 36 years.

Only one of the two *TH* mutations that we found in our study had the potential to change protein structure: the Gly397Arg mutation changed a neutral and polar glycine to a basic arginine. We have not found homozygous *TH* mutations in any of the DRD patients. So far, only autosomal recessive mode of *TH* inheritance was found in DRD patients [6], no disease-causing heterozygous *TH* mutation was reported. Therefore, these two *TH* sequence variants are unlikely to be disease-causing mutations.

We failed to find *GCH1* or *TH* mutations in two affected sisters (**Table 1**, family 2) and eight sporadic cases. Furukawa and Kish [20] reported that almost 40% of DRD patients had no *GCH1* coding region mutations, which was also the case in our study. Thus, although *GCH1* and/or *TH* mutations may indicate DRD, many DRD subjects may be *GCH1*/*TH* mutation free. Alternatively, other related diseases, such as juvenile parkinsonism, may account for the *GCH1* mutation-free DRD cases [13]. The diagnosis of DRD is mainly based on the patient’s symptoms and reaction to treatment, so it is possible that in some cases Parkinson-like symptoms may be misclassified as DRD.

Recently, several studies found deletions on either *GCH1* exons or the promoter region in DRD patients [21]–[28]. In DRD patients without missense or exon-intron boundary mutations, large deletions could be found in 68% of these individuals [28]. Although we have not screened our subjects for deletions in this study, it is possible that deletions may account for the genetic background of *GCH1* mutation-free subjects.

The dopamine synthesis pathway is complex and has profound effects on movement disorders. We have identified three novel *GCH1* mutations in DRD pedigrees and two *TH* mutations in DRD subjects. For the “mutation-free” individuals, it is necessary to screen either deletions in the *GCH1* gene, more genes in the dopamine pathway, Parkinson-related genes, or possibly even the whole genome, to identify the complete genetic background of DRD.

## Supporting Information

Table S1 **Primers used in GTP cyclohydrolase I (** ***GCH1*** **) and tyrosine hydroxylase (** ***TH*** **) PCR sequencing.**
(DOCX)
