# Supplementary material for: GTP Cyclohydrolase I and Tyrosine Hydroxylase Gene Mutations in Familial and Sporadic Dopa-Responsive Dystonia Patients
Source: PLoS One. 2013 Jun 6;8(6):e65215. doi: 10.1371/journal.pone.0065215 (PMC3675154; doi:10.1371/journal.pone.0065215)
Supplement: Table S1 — Primers used in GTP cyclohydrolase I ( GCH1 ) and tyrosine hydroxylase ( TH ) PCR sequencing. (DOCX) [file pone.0065215.s001.docx]

**Table S1**. Primers used in GTP cyclohydrolase I (*GCH1*) and tyrosine hydroxylase (*TH*) PCR sequencing

| Gene/exon | Left primer | Right primer |
| --- | --- | --- |
| *GCH1* |  |  |
| Exon 1 | CTGGACGACTGCCTCTTTTC | GTGAGGCAACTCCGGAAACT |
| Exon 2 | TTTTAGGGAGGATTAACGTTCG | TTGCTGGGAAACAACAAAGA |
| Exon 3 | TGATTTCCGTCTCTTCTCTGAA | GAGAAGGAAGGGGCATCTGT |
| Exon 4 | GCTTCCAGCTGTTTGTGTCA | TCAGTTGTGTGGCATCACCT |
| Exon 5 | AGCCCACTTGCTTCAACAAT | CCTGGGTGACAGAGCAAGAC |
| Exon 6 | CCAAACCAGCAGCTGTCTAC | GACCGGACAGACAGACAATG |
| *TH* |  |  |
| Exon 1 | CTGGGGAGTGAAGGCAATTA | CCAACAGGGACTCAAACACC |
| Exon 2 | CATTTCCAGGTACCTTCTCAGG | AGGGTTGTGGAACATGAAGG |
| Exon 3 | GGTCTCAGCAGGTGGAGGAG | GGACACGAAGGCCACCAG |
| Exon 4 | TTCCTGCTTTTGCTCCCTAA | CCAGCCTCTCAAGGTCATTT |
| Exon 5/6 | TCTCTTCCTCCATCACCCATA | CTGCAGGACGGAGTCTGG |
| Exon 7 | CTCCTGCCCTTCTCACTCC | CTCTTCTTCCCGGCCTTAGT |
| Exon 8/9/10 | ACTGGGGTGGGGCATTAG | AGCAGGCAGCACACTTCAC |
| Exon 11/12 | CTCCCCATCCTTCATCCTC | GGCTGCAGCAAGGAGAGACT |
| Exon 13 | TGGAGTCAGTGATGCCATTG | CTCAAGGCCAGAAGGAAGG |
| Exon 14 | AGTTGACGAGGGCTCTGC | GGGACAGTGCAGGACCAG |
